# Soluble PD-L1 as a Predictor of the Response to EGFR-TKIs in Non-small Cell Lung Cancer Patients With *EGFR* Mutations

**DOI:** 10.3389/fonc.2020.01455

**Published:** 2020-08-25

**Authors:** Yijun Jia, Xuefei Li, Chao Zhao, Shengxiang Ren, Chunxia Su, Guanghui Gao, Wei Li, Fei Zhou, Jiayu Li, Caicun Zhou

**Affiliations:** ^1^Department of Medical Oncology, Shanghai Pulmonary Hospital and Thoracic Cancer Institute, Tongji University School of Medicine, Shanghai, China; ^2^Department of Lung Cancer and Immunology, Shanghai Pulmonary Hospital, Tongji University School of Medicine, Shanghai, China

**Keywords:** soluble PD-L1, non-small cell lung cancer, EGFR-TKIs, efficacy, prediction

## Abstract

Programmed cell death ligand 1 (PD-L1) expressed on tumor tissues is a vital molecule for immune suppression and its impact on the response to epidermal growth factor receptor tyrosine kinase inhibitors (EGFR-TKIs) has been reported. The significance of soluble PD-L1 (sPD-L1) for lung cancer patients remains unknown. This study investigated whether sPD-L1 could predict the response of *EGFR*-mutated non-small cell lung cancer (NSCLC) to EGFR-targeted therapy. We retrospectively evaluated patients who received first-line treatment with EGFR-TKIs for advanced NSCLC with *EGFR* mutations. Pre-treatment plasma concentrations of PD-L1 and on-treatment (1 month after treatment initiation) plasma concentrations of PD-L1 were measured using the R-plex Human PD-L1 assay. The association between the sPD-L1 level and the clinical outcome was analyzed. Among 66 patients who were eligible for the study, patients with high pre-treatment or on-treatment sPD-L1 levels had decreased objective response rate (ORR) compared with that of patients with low sPD-L1 levels (39.4 vs. 66.7%, *p* = 0.026 for pre-treatment sPD-L1 level, and 43.5 vs. 73.9%, *p* = 0.025 for on-treatment sPD-L1 level). A high baseline sPD-L1 level was associated with a shortened progression-free survival (PFS) rate (9.9 vs. 16.1 months, *p* = 0.005). Both univariate and multivariate analyses showed that a high baseline sPD-L1 level was an independent factor associated with the PFS (hazard ratio [HR] 2.56, *p* = 0.011). Our study revealed that the sPD-L1 level was strongly related to the outcome of EGFR-TKIs in NSCLC patients harboring *EGFR* mutations.

## Introduction

Lung cancer remains the leading cause of cancer-related death around the world. Despite significant improvements in the treatment of this malignancy, the prognosis remains poor (1). In recent decades, targeted therapies such as the EGFR-TKIs have markedly improved the management of NSCLC patients with *EGFR* mutations (2–4). Nevertheless, in most patients, the disease inevitably progresses despite an initial dramatic and rapid response to the EGFR-TKIs. Some patients demonstrate a primary resistance to EGFR-TKIs in spite of harboring *EGFR*-sensitive mutations (5). Preclinical studies have suggested that the immune microenvironment can influence the effects of targeted therapy and may serve as one of the mechanisms of resistance to small molecule inhibitors (6–8), but the clinical significance of this interaction in *EGFR*-mutant NSCLC has not been well-verified.

The activation of the programmed cell death protein 1/programmed cell death ligand 1 (PD-1/PD-L1) pathway, which leads to exhausted T-cells and continuous cancer growth, has been identified as the most critical mechanism of tumor evasion (9). PD-1/PD-L1 antibodies have demonstrated impressive anti-tumor responses by releasing the PD-1/PD-L1-mediated control of the immune system, and this activity has therefore become a highly promising treatment strategy for NSCLC in recent years (10). However, NSCLC patients with *EGFR* mutations exhibited a rather low response to PD-1/PD-L1 checkpoint inhibitors (11). Recent studies have identified the association between upregulation of the PD-1/PD-L1 pathway and a resistance to EGFR-targeted therapy. Han et al. detected increased PD-L1 expression when patients acquired a resistance to EGFR-TKIs (12). High levels of PD-L1 expression were also reported to be correlated with a primary resistance and predicted a poor response to EGFR-TKIs (13, 14). These findings may provide implications for using PD-1/PD-L1 inhibitors in patients with *EGFR*-mutant NSCLC.

Evaluating PD-L1 expression in tumor tissue is challenging. First, it is not easy to obtain sufficient tumor samples for analysis from inoperable patients. Furthermore, the test results of PD-L1 expression may differ according to the anti-PD-L1 antibodies applied (15). And the results may vary due to the intra-tumor heterogeneity of PD-L1 expression (16). Soluble forms of PD-L1 (sPD-L1) have recently been identified in blood samples of patients with various malignancies (17–21). A previous study has shown that sPD-L1 may impair host immunity and contribute to systemic immunosuppression, subsequently leading to cancer progression and a poor clinical outcome (22). In lung cancer, it has been reported that high sPD-L1 levels in plasma were associated with a poor prognosis (18). The association between sPD-L1 level and clinical outcome of EGFR-TKIs have not been elaborated, however. Therefore, our study aimed to investigate the impact of sPD-L1 levels on the treatment response to EGFR-TKIs in treatment-naïve NSCLC patients with *EGFR* mutations.

## Materials and Methods

### Study Population

For this retrospective study, we included patients with advanced NSCLC who had started EGFR-TKI treatment between 2014 and 2016 at the Shanghai Pulmonary Hospital. The inclusion criteria were a diagnosis of histologically or cytologically confirmed NSCLC, a sensitizing *EGFR* mutation (defined as *19DEL* or *L858R*), a treatment-naïve status regarding EGFR-TKIs and a thorough documentation of the response evaluation for patients. The treatment response was evaluated every 2–3 months using computerized tomography according to the Response Evaluation Criteria in Solid Tumors version 1.1. (23) Clinicopathological characteristics including gender, age, Eastern Cooperative Oncology Group (ECOG) performance status (PS), histological type, presence of metastases, *EGFR* mutation status and smoking status, were obtained by a review of medical records. The study was approved by the Ethics Committee of the Shanghai Pulmonary Hospital and was conducted according to the Declaration of Helsinki.

### Blood Samples

Blood samples were collected in EDTA tubes prior to the initiation of EGFR-TKI treatment and after 1 month of such treatment. Plasma samples were isolated by centrifugation and stored at −80°C until use. All experiments followed the standard biosecurity and safety procedures of Shanghai Pulmonary Hospital.

### Determination of Soluble PD-L1 Levels

The plasma sPD-L1 level was measured using the R-plex Human PD-L1 kit from Meso Scale Discovery (Rockville, MD, USA) according to the manufacturer's instructions. All the samples were tested in duplicate.

### Statistical Analysis

Continuous data were summarized as medians and ranges. When assessing changes in sPD-L1 levels, for each patient with available blood sample, we estimated the difference between levels at baseline and at 1 month after initiating an EGFR-TKI. Patients with a change in sPD-L1 level that was lower than the median difference for the entire population were considered to have a reduction in sPD-L1 levels, whereas others were considered to have no reduction in sPD-L1 levels. For pre-treatment and on-treatment sPD-L1 levels, values that were lower than the median concentration for the entire population were considered to be low, whereas those above or equal to the median concentration were considered to be high.

The relationship between categorical parameters was determined using a chi-square test or Fisher's exact test. The student's *t*-test or Mann-Whitney *U*-test was used for comparing continuous data according to the data distribution determined by the Kolmogorov-Smirnov test. Kaplan-Meier curves and the log-rank test were used to compare survival times across different patient groups. The Cox proportional hazards regression analysis was performed, and HRs and 95% confidence intervals (CIs) were calculated to determine the survival difference. Variables were included in the multivariate analysis if they were statistically significant (*p* < 0.10) in the univariate analysis. All statistical analyses were performed using GraphPad Prism software (version 8; GraphPad, Inc., LaJolla, CA) and SPSS statistical software (version 22.0; IBM Corporation, Armonk, NY). Results were considered statistically significant at a two-sided *p* < 0.05.

## Results

### Distribution of Plasma sPD-L1 and Patient Characteristics

In total, 66 patients met the inclusion criteria and were enrolled in this study. On-treatment blood samples were collected for 46 of these patients. The distributions of pre-treatment and on-treatment plasma sPD-L1 concentrations are shown in Table 1. The median pre-treatment and on-treatment sPD-L1 levels were 568.19 pg/ml (range: 344.96–1889.49 pg/ml) and 560.99 pg/ml (range: 305.13–2255.57 pg/ml), respectively. The median difference among the pre-treatment and on-treatment sPD-L1 concentrations was 6.88 pg/ml (range: −454.08–743.72 pg/ml). The median % change in the pre-treatment and on-treatment sPD-L1 level was 19.19% (range: 0.5–116.61%).

**Table 1:** Distribution of plasma soluble PD-L1 concentration.

**Variable**	**Concentration (pg/ml)**
**Plasma sPD-L1 Level (Pre-treatment)**	
Median (Range)	568.19 (344.96–1889.49)
**Plasma sPD-L1 Level (On-treatment)**	
Median (Range)	560.99 (305.13–2255.57)
**Difference Among sPD-L1 Level**	
Median (Range)	6.88 (−454.08–743.72)
**% Change in sPD-L1 Level**	
Median (Range) (%)	19.19 (0.5–116.61)

The demographic and clinical characteristics are demonstrated in Table 2. Of the 66 patients, 30 (45.4%) were female and 36 (54.5%) were male. The median age was 61. Most patients were diagnosed with adenocarcinoma (93.9%, *n* = 62) and had an ECOG PS status of 0–1 (97.0%, *n* = 64). Fifty-six patients (84.8%) were at stage IIIB to IV at the time of diagnosis, and 10 patients (15.2%) were with recurred disease. A majority of patients were non-smokers (75.8%, *n* = 50), and 16 patients (24.2%) were current or former smokers. Regarding the baseline *EGFR* mutation status, 33 patients (50.0%) harbored the *exon 19 deletion* and 33 patients (50.0%) had the *exon 21 L858R* point mutation (of this latter group, one patient had a co-mutation of *L858R* and *L861Q*). A majority of patients were treated with first-line EGFR-TKIs (gefitinib: *n* = 49, icotinib: *n* = 13, erlotinib: *n* = 2) and two patients received afatinib treatment. As for the metastasis status, there were 22 patients (33.3%) with brain metastases, 24 patients (36.4%) with bone metastases, and four patients with liver metastases at diagnosis. Among all patients, 38 patients received the *EGFR T790M* test at progression; 22 of these patients harbored an *EGFR T790M* mutation when they became resistant to first-line EGFR-TKIs.

**Table 2:** Patient characteristics.

**Variable**	**All** ***N* = 66 (%)**	**Low sPD-L1** ***N* = 33 (%)**	**High sPD-L1** ***N* = 33 (%)**	***P*-value**
**Gender**				
Female	30 (45.4)	16 (48.5)	14 (42.4)	0.621
Male	36 (54.5)	17 (51.5)	19 (57.6)	
**Age (years)**				
Range	35–84	43–76	35–84	0.054
Median	61	55	63	
**Histology**				
Adenocarcinoma	62 (93.9)	31 (93.9)	31 (93.9)	1.000
NSCLC-NOS	4 (6.1)	2 (6.1)	2 (6.1)	
**ECOG PS**				
0–1	64 (97.0)	33 (100.0)	31 (93.9)	0.473
2	2 (3.0)	0 (0.0)	2 (6.1)	
**Stage**				
Recurrence	10 (15.2)	4 (12.1)	6 (18.2)	0.492
IIIB-IV	56 (84.8)	29 (87.9)	27 (81.8)	
**Smoking**				
Never	50 (75.8)	25 (75.8)	25 (75.8)	1.000
Current/former	16 (24.2)	8 (24.2)	8 (24.2)	
**EGFR Status**				
19DEL	33 (50.0)	20 (60.6)	13 (39.4)	0.085
L858R and others	33 (50.0)	13 (39.4)	20 (60.6)	
**TKIs**				
Gefitinib	49 (74.2)	24 (72.7)	25 (75.8)	0.207
Erlotinib	2 (3.0)	2 (6.1)	0 (0.0)	
Icotinib	13 (19.7)	5 (15.2)	8 (24.2)	
Afatinib	2 (3.0)	2 (6.1)	0 (0.0)	
**Brain Metastasis**				
Yes	22 (33.3)	10 (30.3)	12 (36.4)	0.602
No	44 (66.7)	23 (69.7)	21 (63.6)	
**Bone Metastasis**				
Yes	24 (36.4)	9 (27.3)	15 (45.5)	0.125
No	42 (63.6)	24 (72.7)	18 (54.5)	
**Liver Metastasis**				
Yes	4 (6.1)	2 (6.1)	2 (6.1)	1.000
No	62 (93.9)	31 (93.9)	31 (93.9)	
**T790M Detected at Progression**				
Yes	22 (33.3)	11 (33.3)	11 (33.3)	1.000
No	16 (24.2)	8 (24.2)	8 (24.2)	

*NSCLC-NOS, non-small cell lung cancer-not otherwise specified; ECOG PS, Eastern Cooperative Oncology Group performance status*.

*L858R and others, one patient had a L858R and L861Q co-mutation*.

*P-values are calculated using Chi-square test or Fisher's exact test. Student' t-test was used for age. Bolded p-values indicate significance*.

The patient cohort was divided into two groups based on the level of sPD-L1 before treatment had been initiated. There were no significant differences in gender, age, histological status, ECOG PS status, stage, smoking status, *EGFR* mutation status, type of EGFR-TKI treatment received, metastasis status or *T790M* mutation at progression between the low sPD-L1 expression group and high sPD-L1 expression group.

### High sPD-L1 Expression Is Associated With a Poor Response to EGFR-TKIs

Clinical characteristics of patients and distributions of sPD-L1 concentrations according to the therapeutic response to EGFR-TKIs are listed in Table 3. The ORR among the whole cohort was 53.0%, with 35 patients achieving a partial response (PR) and no patient achieving a complete response. The plasma sPD-L1 levels were significantly correlated with the treatment response. Patients with a pre-treatment sPD-L1 level of <568.19 had an obviously higher ORR than those with a pre-treatment sPD-L1 level of more than or equal to 568.19 (66.7 vs. 39.4%, *p* = 0.026). Meanwhile, a higher on-treatment sPD-L1 level was also associated with a poor response to EGFR-TKIs. The ORR was 73.9% in patients with low on-treatment sPD-L1 levels, but the ORR was only 43.5% in patients with high on-treatment sPD-L1 levels. There were no differences in the treatment response between patients with or without a reduction of sPD-L1 levels. Other clinical characteristics, including gender, age, ECOG PS score, tumor stage, smoking status, *EGFR* status, and type of EGFR-TKI received were not associated with the therapeutic response.

**Table 3:** Clinical characteristics of patients and distributions of sPD-L1 concentrations according to the therapeutic response to EGFR-TKIs.

	**Objective response rate (ORR)**		***P***
	**Yes** ***N* = 35**	**No** ***N* = 31**	
**Gender**			
Female	46.7 (14/30)	53.3 (16/30)	0.344
Male	58.3 (21/36)	41.7 (15/36)	
**Age**			
<61	63.6 (21/33)	36.4 (12/33)	0.084
≥61	42.4 (14/33)	57.6 (19/33)	
**ECOG PS**			
0–1	54.7 (35/64)	45.3 (29/64)	0.217
2	0.0 (0/2)	100.0 (2/2)	
**Stage**			
Recurrence	40.0 (4/10)	60.0 (6/10)	0.581
IIIb/IV	55.4 (31/56)	44.6 (25/56)	
**Smoking**			
Never	50.0 (25/50)	50.0 (25/50)	0.383
Current/former	62.5 (10/16)	37.5 (6/16)	
**EGFR Status**			
19DEL	57.6 (19/33)	42.4 (14/33)	0.459
L858R and others	48.5 (16/33)	51.5 (17/33)	
**TKIs**			
Gefitinib	53.1 (26/49)	46.9 (23/49)	0.734
Erlotinib	50.0 (1/2)	50.0 (1/2)	
Icotinib	46.2 (6/13)	53.8 (7/13)	
Afatinib	100.0 (2/2)	0.0 (0/2)	
**Brain Metastasis**			
Yes	63.6 (14/22)	36.4 (8/22)	0.222
No	47.7 (21/44)	52.3 (23/44)	
**Plasma sPD-L1 Levels (Pre-treatment)**			
<568.19	66.7 (22/33)	33.3 (11/33)	**0.026**
≥568.19	39.4 (13/33)	60.6 (20/33)	
**Plasma sPD-L1 Levels (On-treatment)**			
<560.99	73.9 (17/23)	26.1 (6/23)	**0.025**
≥560.99	43.5 (10/23)	56.5 (13/23)	
**Plasma sPD-L1 Reduction**			
Yes	60.9 (14/23)	39.1 (9/23)	0.765
No	56.5 (13/23)	43.5 (10/23)	
**T790M Detected at Progression**			
Yes	68.2 (15/22)	31.8 (7/22)	0.132
No	43.8 (7/16)	56.2 (9/16)	

*NSCLC-NOS, non-small cell lung cancer-not otherwise specified; ECOG PS, Eastern Cooperative Oncology Group performance status*.

*L858R and others, one patient had a L858R and L861Q co-mutation*.

*P-values are calculated using Chi-square test or Fisher's exact test. Bolded p-values indicate significance*.

**Figure 1:**
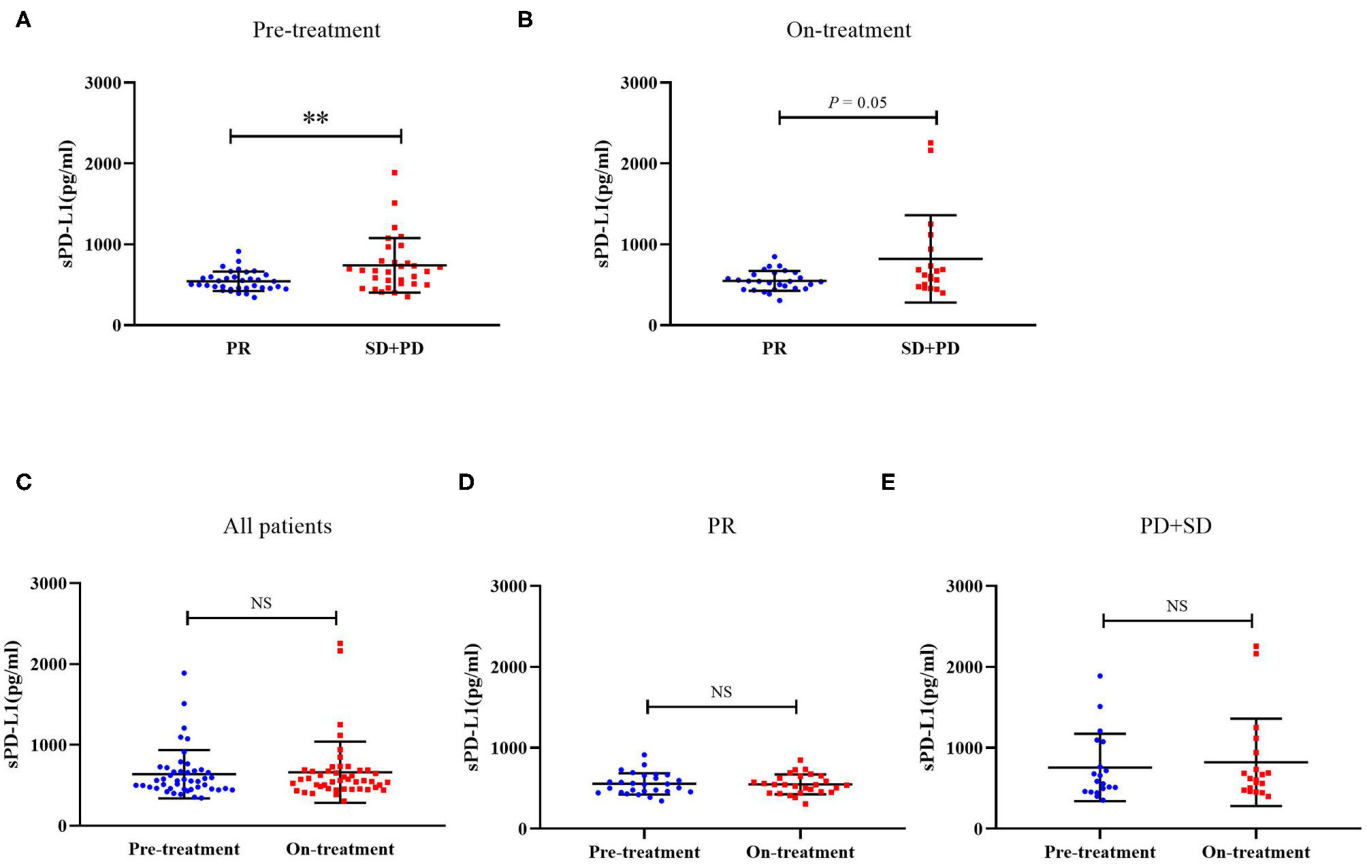
**(A)** Distribution of sPD-L1 concentrations prior to the initiation of EGFR-TKI treatment according to therapeutic responses. **(B)** Distribution of sPD-L1 concentrations 1 month after initiating EGFR-TKI treatment according to therapeutic responses. **(C)** sPD-L1 concentrations at pre-treatment and on-treatment in whole patient group. **(D)** sPD-L1 concentrations at pre-treatment and on-treatment in patients with PR. **(E)** sPD-L1 concentrations at pre-treatment and on-treatment in patients with SD or PD (Results are presented as mean ± SD. **P < 0.01; NS, Not Significant).

We next compared both the pre-treatment and on-treatment sPD-L1 concentrations in patients who achieved a PR and patients who had a best response of stable disease (SD) or progressive disease (PD). The PR group demonstrated significantly lower levels of pre-treatment plasma sPD-L1 compared with the SD+PD group. As for the on-treatment plasma sPD-L1 levels, although the finding was marginally significant, the PR group also showed a lower level of sPD-L1. In whole patient group and subgroups divided by treatment response, the levels of sPD-L1 were not significantly changed by EGFR-TKIs treatment (Figure 1).

### Lower Levels of sPD-L1 Before EGFR-TKI Initiation Are Associated With Improved Survival Rates

**Figure 2:**
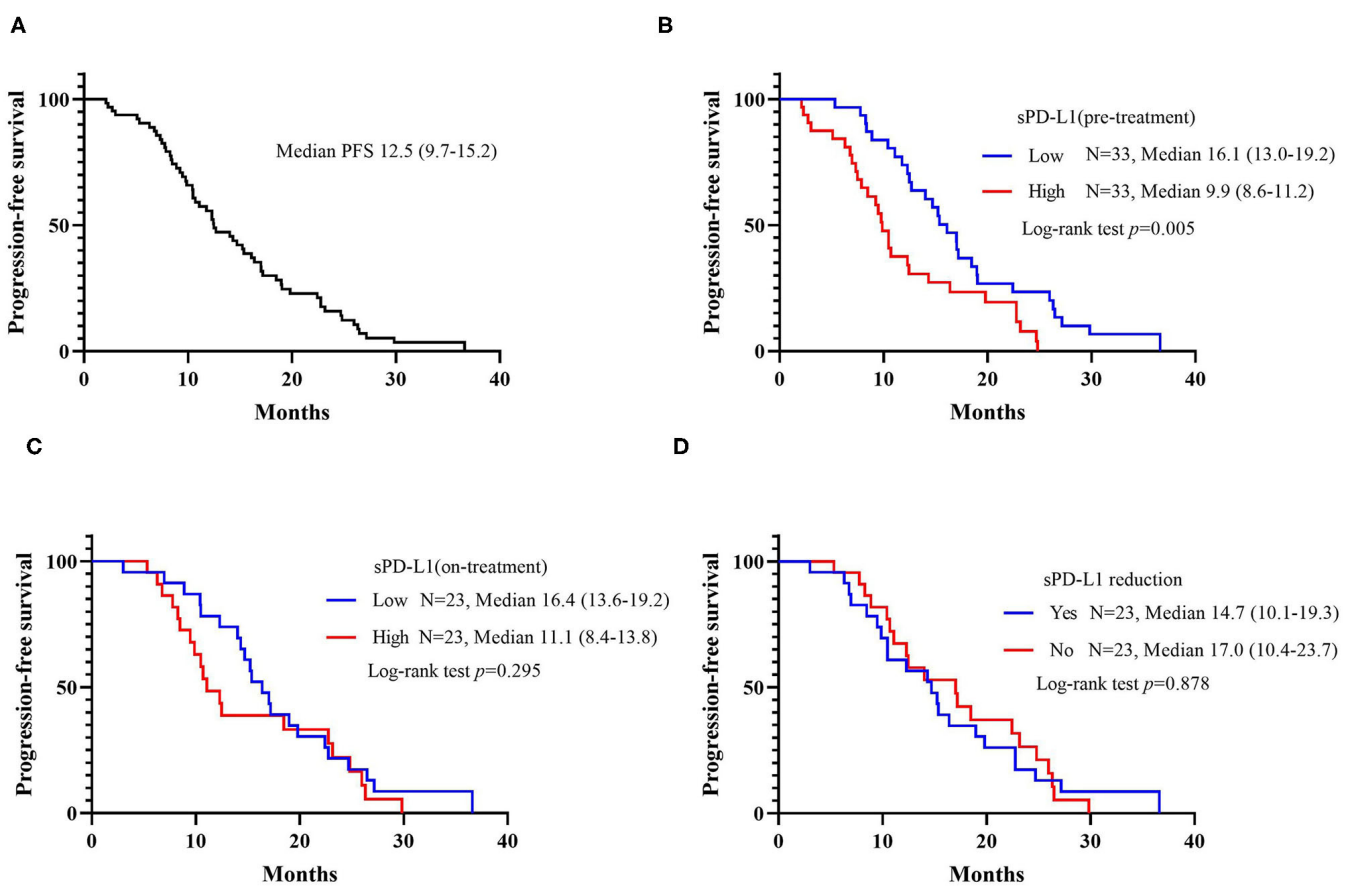
Kaplan-Meier curves for progression-free survival (PFS). **(A)** PFS among all patients. **(B)** PFS according to the baseline level of sPD-L1. **(C)** PFS according to the sPD-L1 levels 1 month after initiating EGFR-TKI treatment. **(D)** PFS according to the changes in sPD-L1 during EGFR-TKI treatment.

The median progression-free survival (PFS) in the whole patient group was 12.5 months (95% CI: 9.7–15.2 months). As demonstrated in Figure 2, patients with a lower level of pre-treatment sPD-L1 had a statistically superior PFS rate compared with patients with higher pre-treatment sPD-L1 levels. The median PFS was 16.1 months (95% CI: 13.0–19.2 months) vs. 9.9 months (95% CI: 8.6–11.2 months), and the log-rank *p*-value was 0.005. Although it was not statistically significant, a shorter PFS rate was also observed in patients with higher on-treatment sPD-L1 concentrations (median PFS, 11.1 vs. 16.4 months). The change in sPD-L1 levels was not correlated with the PFS rate of patients treated with EGFR-TKIs, however.

To further evaluate the potential impact of clinical variables on the therapeutic efficacy of treatment with first-line EGFR-TKIs, we performed both univariate and multivariate analyses on the whole patient cohort. Typical factors of age, sex, smoking history, stage, *EGFR* driver mutation type, brain metastasis status, sPD-L1 concentration, and *T790M* status at progression were included in the Cox regression analysis. A worse outcome for patients with high sPD-L1 levels before EGFR-TKI treatment was also found for the PFS rate in the Cox regression model with an HR of 2.56 (95% CI: 1.24–5.27, *p* = 0.011). No clinicopathological factors were associated with the PFS rate (Table 4), but the emergence of the *T790M* resistance mutation at progression was correlated with a better PFS rate (HR = 0.45, 95% CI: 0.22–0.94, *p* = 0.033).

**Table 4:** Univariate and multivariate analysis of the clinical factors associated with progression-free survival.

**Variable**	**Progression-free survival**	
	**Univariate analyses**	**Multivariate analyses**
	**HR (95% CI)**	**HR (95% CI)**
	***P***	***P***
Age:	1.15 (0.68–1.94)	
≥61 vs. <61	0.605	
Sex:	1.04 (0.62–1.76)	
male vs. female	0.881	
Smoking:	1.30 (0.71–2.39)	
current/former vs. never	0.391	
Stage:	1.35 (0.66–2.76)	
IIIB-IV vs. recurrence	0.412	
EGFR status:	1.36 (0.80–2.30)	
L858R and others vs. 19DEL	0.254	
Brain metastasis:	1.29 (0.74–2.23)	
Yes vs. No	0.371	
sPD-L1 level (pre-treatment):	2.15 (1.24–3.74)	2.56 (1.24–5.27)
≥568.19 vs. <568.19	**0.007**	**0.011**
sPD-L1 level (on-treatment):	1.39 (0.75–2.57)	
≥560.99 vs. <560.99	0.299	
sPD-L1 reduction:	0.95 (0.51–1.77)	
Yes vs. No	0.879	
T790M detected at progression:	0.55 (0.28–1.10)	0.45 (0.22–0.94)
Yes vs. No	**0.089**	**0.033**

*HR, hazard ratio; CI, confidence interval*.

*Bolded p-values indicate significance. Independent variables with p < 0.10 in the univariate analyses were included in the model*.

## Discussion

A growing number of studies have demonstrated that sPD-L1 might play a crucial role in the prediction of the treatment response of PD-1/PD-L1 inhibitors and also the prognosis of cancer patients (17, 20, 21, 24). However, the significance of the sPD-L1 level in predicting the response to EGFR-TKIs in NSCLC patients remains unclear. The results of the present study revealed that the ORR for first-line EGFR-TKI treatment was higher in *EGFR*-mutant NSCLC patients with low plasma sPD-L1 levels than in those with high sPD-L1 levels. Furthermore, a prolonged PFS rate was significantly associated with a lower pre-treatment sPD-L1 level. Our results suggested that the plasma PD-L1 concentration could be a promising marker for determining the efficacy of EGFR-TKIs for NSCLC patients harboring *EGFR* mutations.

The underlying mechanisms of generation and regulation of the soluble forms of PD-L1 are still unclear. One possible source is spliced variant. Zhou et al. showed that alternative splicing of PD-L1 occurred in all melanoma cell lines and splice variants could result in the secretion of sPD-L1 (21). Besides, it has been reported that tumor-derived extracellular vesicles including exosomes carried PD-L1 on their surfaces (25). Chen et al. demonstrated in their study that sPD-L1 could also be produced through proteolytic cleavage of membrane-bound proteins because the release of sPD-L1 was decreased after tumor cells were treated with the inhibitor of matrix metalloproteinase (26). Frigola et al. reported that the tumor stage and the presence of aggressive pathological features were associated with sPD-L1 levels in renal cell carcinoma, suggesting that circulating sPD-L1 might be derived from tumor tissue (22). Whether sPD-L1 concentrations are correlated with clinicopathological features such as tumor stage in lung cancer is controversial, however. Cheng et al. reported a positive association between sPD-L1 levels and stages of NSCLC (27). In advanced lung cancer, no obvious difference was identified in clinical stage between the low sPD-L1 and high sPD-L1 groups (18, 24). If most of the circulating PD-L1 is derived from membrane PD-L1 on tumor cells, the levels of sPD-L1 should be elevated with an increase in tumor burden. The fact that the patients involved in our study had advanced or recurrent lung cancer explains why we did not observe any correlation between the initial tumor stage and the sPD-L1 level. It has been reported that concentrations of sPD-L1 in blood samples from healthy donors increased as age grew (26). Interestingly, although it was only marginally significant, our results also revealed that sPD-L1 levels tended to be correlated with the age distribution in NSCLC patients. These data suggested that the level of circulating PD-L1 could be associated with the status of the entire immune system.

The impact of membrane form of PD-L1 on the treatment response and prognosis of NSCLC with EGFR mutations has been identified in recent studies (16, 28, 29). However, the conclusions remain controversial. In a study carried out by Lin et al. of *EGFR*-mutant lung adenocarcinoma patients, PD-L1 represented a favorable biomarker for the response to EGFR-TKIs and outcomes of these patients (28). There were also studies showed that high levels of PD-L1 expression were associated with a primary resistance and inferior response to EGFR-TKIs (13, 14). Because the soluble forms of PD-L1 are believed to be released from the PD-1/PD-L1 interaction site in tumor tissue, it is possible that the level of sPD-L1 may be correlated with membrane PD-L1 expression and also have a predictive or prognostic value. In our study, a higher level of sPD-L1 was significantly correlated with a lower ORR and a shorter PFS in *EGFR*-mutant NSCLC treated with EGFR-TKIs. In a recent study, Meyo et al. demonstrated that levels of sPD-L1 did not correlate with PFS in NSCLC patients with EGFR mutations (30). Several possible explanations of the conflicting results would be the differences in sPD-L1 testing assays, patients' characteristic and the definition of a low or high sPD-L1 level. Further studies should be done to validate the association between EGFR-TKI efficacy and sPD-L1 levels. The sPD-L1 level was not only revealing for targeted therapy; low sPD-L1 levels were also favorable markers for outcomes following chemotherapy and immunotherapy (20, 31, 32). In NSCLC, increasing evidences showed that sPD-L1 levels might represent a novel biomarker for the prediction of the efficacy of immune checkpoint therapy (24, 30, 32). These results supported the hypothesis that sPD-L1 binds to PD-1 on circulating T cells in peripheral blood before cytotoxic T cells reach the tumor site, thus impairing T cell-mediated antitumor immune activity and resulting in a poor treatment response for patients with high sPD-L1 levels.

Pre-clinical studies showed that the concentration of sPD-L1 was positively correlated with the expression of PD-L1 in various tumor cell lines and that sPD-L1 also played an important role in immunosuppression (26, 33). In studies carried out in lymphoma patients, serum sPD-L1 levels significantly correlated with the expression of PD-L1 in lymphoma cells and patients with low sPD-L1 levels demonstrated a favorable clinical outcome (33, 34). In gastric cancer, although serum sPD-L1 levels showed a trend of elevation in patients with high tissue PD-L1 expression, a statistically significance was not observed (20). A recent study performed in soft tissue sarcomas also revealed that there were no obvious differences in sPD-L1 levels between tissue PD-L1 positive group and PD-L1 negative group (35). One possible explanation is the mPD-L1 expression may vary within the same tumor spatially and temporally. It is possible that assessment of PD-L1 expression from a single lesion or at a single time point may cause variability. The generation of sPD-L1 may also explain for the inconsistency of sPD-L1 and tissue PD-L1. Except for the main sources mentioned above, the circulating PD-L1 may also be produced by other sources like immune cells, cell injury, or cell death. The correlation between soluble forms and membrane PD-L1 in NSCLC has not been well-described. It is regrettable that the PD-L1 expression on tumor cells was not tested in our patients and that we could not, therefore, analyze the association between levels of membrane PD-L1 and sPD-L1. Costantini et al. revealed in their study that there was no association observed between IHC positivity of PD-L1 and sPD-L1 concentration at the time of diagnosis in NSCLC (32). Further study needs to be done to identify this correlation in NSCLC patients, especially in patients with *EGFR* mutations.

There have been studies supporting the theory that PD-L1 is a downstream molecule of EGFR signaling and EGFR-TKI could down-regulate PD-L1 expression on NSCLC cells by pathways like IL-6/JAK/STAT3, NKκB, or p-ERK1/2/p-c-Jun (36–38). However, the impact of EGFR-TKI treatment on sPD-L1 levels has not been well-elaborated in NSCLC patients with *EGFR* mutations. In our study, there was no significant change between the baseline and on-treatment sPD-L1 concentration. Similarly, Vecchiarelli et al. demonstrated in their study that sPD-L1 levels were elevated in NSCLC patients who received chemotherapy, but not in those who received treatments like TKIs or immunotherapy (39). There are evidences suggesting that EGFR-TKI may have an immunostimulatory effect by potentiating the induction of antigen presenting proteins in response to interferon-γ and enhancing T cells or NK cells mediated tumor killing (40–43). Considering the production of circulating PD-L1 was reported to be correlated with stimulation with interferon-γ (25), it is understandable that EGFR-TKI treatment did not decrease sPD-L1 levels like membrane PD-L1 on tumor cells do. Also, the sPD-L1 levels at the time when patients developed acquired resistance to EGFR-TKI treatment were not evaluated in this study. It has been reported that the expression of membrane PD-L1 was elevated when patients became resistant to first-line EGFR-TKIs (12). Further studies including a larger patient cohort should be done to verify this phenomenon with sPD-L1.

Emergence of the *T790M* resistance mutation accounts for 50–60% of cases with acquired resistance to first-generation EGFR-TKIs (44). Osimertinib, a third-generation EGFR-TKI that selectively inhibits the *EGFR T790M* mutation, has been a successful treatment for patients with *T790M*-positive NSCLC who have acquired resistance to prior-line EGFR-TKIs (45). However, the underlying mechanism is unknown in many patients with acquired resistance to EGFR-TKIs. Recently, the correlation between membrane PD-L1 expression and *T790M* status after disease progression during EGFR-TKI treatment was reported. It seemed that among *T790M*-negative patients, more demonstrated high levels of PD-L1 expression when they were resistant to first-line EGFR-TKIs (46), making us wonder if PD-L1 expression could represent a novel mechanism of resistance. In our study, although baseline sPD-L1 levels could predict the response to EGFR-TKIs, no significant association was observed between the plasma PD-L1 level and the *T790M* status. The small sample sizes in this study may have had an influence. Only 38 patients had a *T790M* mutation test when they progressed to prior-line EGFR-TKI treatment.

There are several limitations in this present study. First, as a retrospective study, the conclusions generated in our study still need further prospective studies to be confirmed. Second, our study mainly discussed the correlation between sPD-L1 level and response to EGFR-TKI treatment. The influence of sPD-L1 level on overall survival of NSCLC needs to be assessed in further studies. Third, as a study carried out in a single institution, the patient number is relatively small, especially when analyzing patients with secondary *T790M* mutation. Multi-centered study with a larger patient number is needed to verify our results.

In conclusion, this retrospective study revealed that high plasma sPD-L1 levels were associated with poor response to EGFR-TKIs and that this finding could be a promising biomarker in patients with *EGFR*-mutant advanced NSCLC.

## Data Availability Statement

The raw data supporting the conclusions of this article will be made available by the authors, without undue reservation.

## Ethics Statement

The studies involving human participants were reviewed and approved by Ethics Committee of the Shanghai Pulmonary Hospital. The patients/participants provided their written informed consent to participate in this study. Written informed consent was obtained from the individual(s) for the publication of any potentially identifiable images or data included in this article.

## Author Contributions

YJ, XL, and CZho designed this study and drafted the manuscript. YJ, XL, CZha, FZ, and JL reviewed the patient record, collected patient samples, and conducted the relevant experiments. YJ, CZha, GG, and WL performed the statistical analyses. SR and CS provided critical comments and revised the manuscript. All authors read and approved the final version of the manuscript.

## Conflict of Interest

The authors declare that the research was conducted in the absence of any commercial or financial relationships that could be construed as a potential conflict of interest.
